# Tryptophan-metabolizing gut microbes regulate adult neurogenesis via the aryl hydrocarbon receptor

**DOI:** 10.1073/pnas.2021091118

**Published:** 2021-07-01

**Authors:** George Zhang Wei, Katherine A. Martin, Peter Yuli Xing, Ruchi Agrawal, Luke Whiley, Thomas K. Wood, Sophia Hejndorf, Yong Zhi Ng, Jeremy Zhi Yan Low, Janet Rossant, Robert Nechanitzky, Elaine Holmes, Jeremy K. Nicholson, Eng-King Tan, Paul M. Matthews, Sven Pettersson

**Affiliations:** ^a^Lee Kong Chian School of Medicine, Nanyang Technological University, Singapore 636921;; ^b^National Neuroscience Institute, Singapore 169857;; ^c^The Singapore Centre for Environmental Life Sciences Engineering, School of Biological Sciences, Nanyang Technological University, Singapore 637551;; ^d^Interdisciplinary Graduate School, Nanyang Technological University, Singapore 637335;; ^e^Australian National Phenome Centre, Health Futures Institute, Murdoch University, Perth WA 6150, Australia;; ^f^Perron Institute for Neurological and Translational Science, Nedlands WA 6009, Australia;; ^g^Department of Chemical Engineering, Pennsylvania State University, University Park, PA 16802;; ^h^Department of Neurobiology, Care and Society, Karolinska Institutet, 171 77 Stockholm, Sweden;; ^i^The School of Biological Sciences, Nanyang Technological University, Singapore 637551;; ^j^Program in Developmental and Stem Cell Biology, Peter Gilgan Centre for Research and Learning, Hospital for Sick Children, Toronto, ON M5G 0A4, Canada;; ^k^Princess Margaret Cancer Centre, University Health Network, University of Toronto, Toronto, ON M5G 2C1, Canada;; ^l^Section for Nutrition Research, Imperial College London, London SW7 2AZ, United Kingdom;; ^m^Institute of Global Health Innovation, Imperial College London, London SW7 2NA, United Kingdom;; ^n^UK Dementia Research Institute, Imperial College London, London SW7 2AZ, United Kingdom;; ^o^Department of Surgery and Cancer, Imperial College London, London SW7 2AZ, United Kingdom;; ^p^Department of Brain Sciences, Imperial College London, London W12 0NN, United Kingdom;; ^q^Faculty of Medical Sciences, Sunway University, 47500 Kuala Lumpur, Malaysia

**Keywords:** microbiota, tryptophan metabolism, indole, aryl hydrocarbon receptor, neurogenesis

## Abstract

While modulatory effects of gut microbes on neurological phenotypes have been reported, the mechanisms remain largely unknown. Here, we demonstrate that indole, a tryptophan metabolite produced by tryptophanase-expressing gut microbes, elicits neurogenic effects in the adult mouse hippocampus. Neurogenesis is reduced in germ-free (GF) mice and in GF mice monocolonized with a single-gene *tnaA* knockout (KO) mutant *Escherichia coli* unable to produce indole. External administration of systemic indole increases adult neurogenesis in the dentate gyrus in these mouse models and in specific pathogen-free (SPF) control mice. Indole-treated mice display elevated synaptic markers postsynaptic density protein 95 and synaptophysin, suggesting synaptic maturation effects in vivo. By contrast, neurogenesis is not induced by indole in aryl hydrocarbon receptor KO (AhR^−/−^) mice or in ex vivo neurospheres derived from them. Neural progenitor cells exposed to indole exit the cell cycle, terminally differentiate, and mature into neurons that display longer and more branched neurites. These effects are not observed with kynurenine, another AhR ligand. The indole-AhR–mediated signaling pathway elevated the expression of *β-catenin*, *Neurog2*, and *VEGF-α* genes, thus identifying a molecular pathway connecting gut microbiota composition and their metabolic function to neurogenesis in the adult hippocampus. Our data have implications for the understanding of mechanisms of brain aging and for potential next-generation therapeutic opportunities.

## Significance

While the effects of gut microbes on brain development and function have been described, the mechanisms remain largely unknown. Here, we report that tryptophan-metabolizing gut microbes secrete indoles that regulate neurogenesis in the adult hippocampus. This stimulatory effect on adult neurogenesis is mediated by the metabolic- and immune-linked aryl hydrocarbon receptor (AhR). Another AhR ligand, the tryptophan metabolite kynurenine, failed to induce neurogenesis, suggesting ligand specificity of AhR-mediated regulation of adult neurogenesis. The indole-AhR signaling pathway elevates transcription factors and signaling proteins that promote adult neurogenesis, as well as key markers of synaptic maturation. Our data demonstrate a symbiotic gut–brain coregulatory axis that connects the metabolic status of gut microbes to the control of neurogenesis in the adult hippocampus.

The emergence of nerve cells was a major evolutionary transition required for the formation of multicellular life and, remarkably, predates the emergence of the mesoderm. That is, nerve cells appeared before the mesoderm layer, which, among many different cell types, control blood cells and adaptive immunity (1). Neural stem and intermediate progenitor cells reside in specialized niches of the adult mammalian brain and give rise to new neurons throughout life. In contrast to neurogenesis in early life that requires appropriate stimulation at “critical periods” in development to establish functional neuronal circuits (2, 3), adult hippocampal neurogenesis (AHN) requires continuous stimulation throughout life (4). The current view holds that AHN functionally contributes to learning and memory (5–7) as well as regulating the hypothalalmic–pituitary–adrenal (HPA) axis in response to stress (8). Adult neural stem cells (NSCs) largely reside in a mitotically dormant, quiescent state (9) but can be activated and respond to interventions, including physical exercise and diet (10–14). Therefore, it has been suggested that new neurons are generated “on demand” in response to environmental stimuli or stressors (10). This raises the interesting prospect that AHN may have conferred evolutionary advantages to mammals, for example, in mediating a metabolic stress trigger of food-seeking behavior for survival. Mechanisms by which environmental signals regulate adult neurogenesis are incompletely understood, although circulating hormones and growth factors including adiponectin brain-derived neurotrophic factor and vascular endothelial growth factor (VEGF) have been implicated (14–22).

Animal evolution and cell fate specification, including cells of the nervous system, are influenced by the presence of microbes. Following initial observations that gut microbes affect the postnatal development of the HPA axis stress response (23) and alter host brain development and anxiety behaviors of germ-free (GF) mice devoid of microbes (24, 25), gut microbes have rapidly attracted attention for their roles in gut-to-brain communication [reviewed by Kundu, Blacher, Elinav, and Pettersson (26)]. Indeed, several hippocampal-dependent behaviors are included in the growing repertoire of functions linked to microbes, including fear extinction and anxiety-like responses (23, 24, 27–29).

Gut microbes are an evolving, prokaryotic component of the metaorganismal self. They secrete myriad metabolites of which several are known to regulate cell function and the integrity of permeable barriers, including the blood–brain barrier (BBB) (30, 31). Importantly, the microbial metabolic output is dynamic, allowing microbes to respond to environmental cues, including nutritional fluctuation. Indoles are microbial metabolites of dietary tryptophan that are produced in response to conditions of low glucose availability and act to adaptively inhibit microbial replication (32–34). Indeed, diet appears to be a major regulator of gut microbiota composition and function (35, 36). An increasing range of observations suggest that changes in the gut microbiota may influence the brain. GF rodents exhibit elevated tryptophan levels and reduced indole derivatives in serum (37–39). Functionally, an association between reduced serum indoxyl sulfate (a liver metabolite of indole) in GF or antibiotic-treated mice and impaired fear extinction learning has been reported (27). Translationally, the administration of indole, indole derivatives, or microbial tryptophanase enzyme to antibiotic-treated experimental autoimmune encephalomyelitis mice—a multiple sclerosis model system—reduced CNS inflammation and improved disease scores by the activation of the aryl hydrocarbon receptor (AhR) pathway in astrocytes (40). In humans, while several studies have associated diseases of the CNS with altered tryptophan metabolism (41–44), the clinical potential of indole remains to be elucidated.

The aryl hydrocarbon receptor (AhR) is a ligand-induced transcription factor that, upon binding a cognate ligand, heterodimerizes with the AhR nuclear translocator (ARNT) and translocates to the nucleus to activate downstream target genes. Different ligands are thought to induce particular conformational changes of the AhR/ARNT heterodimer complex, which determines the recruitment of cofactors that establish downstream enhancer and promoter gene activation. Interestingly, the AhR is also a target for tryptophan metabolites, including microbiota-derived indoles and eukaryotic kynurenines [reviewed by Hubbard, Murray, and Perdew (45)]. AhR-dependent pathways also influence host–microbe interactions. For example, in the intestine, AhR modulates the gut microbiota community structure in mice (46, 47), immune activity, and maintenance of the epithelial barrier function (48). Recent data describe how AhR-dependent pathways are important for a wide range of biological functions, including development, metabolic homeostasis, cell growth, and differentiation in multiple tissues, including those of the central nervous system [reviewed by Lee and McPherson (49)].

The spatiotemporal expression of AhR messenger RNA (mRNA) in the developing embryonic brain as well as the juvenile and adult mouse hippocampus suggests a role for this signaling pathway and transcriptional activator in regulating neurogenesis across the lifespan (50). In the adult brain, the AhR is localized in neural progenitor cells (NPCs), granule cells, and astrocytes of the dentate gyrus (DG), where its signaling has been associated with NPC proliferation, fate specification, and dendritic development (51–56). Moreover, deletion or 2,3,7,8-tetrachlorodibenzodioxin (TCDD) activation of the AhR diminishes NPC proliferation, neuronal differentiation, and impairment of contextual fear memory behavior in mice (51). These results suggest a link between tryptophan-metabolizing gut microbes, AhR signaling, and adult neurogenesis. Here, we report the identification of a gut microbe-indole-AhR–mediated signaling pathway that regulates neurogenesis both in the adult mouse hippocampus in vivo and in ex vivo neurosphere cultures.

## Results

### Gut Microbiota-Derived Indoles Regulate Adult Neurogenesis.

**Fig. 1.**
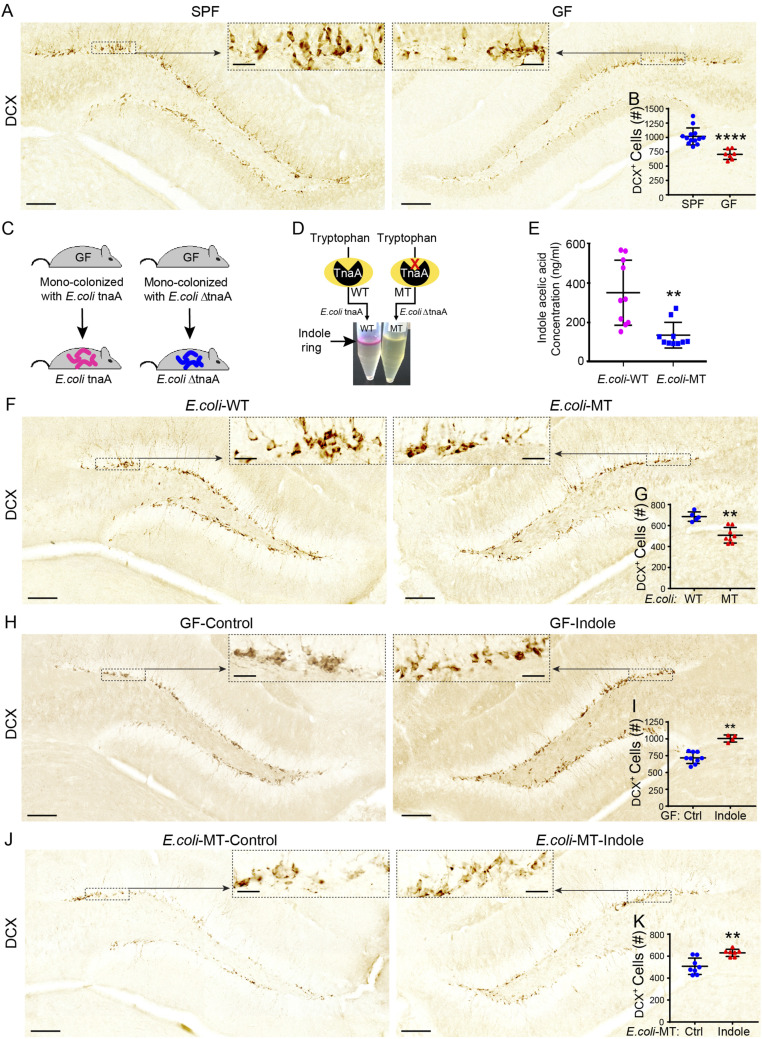
Exposure to indole rescues neurogenesis deficits in “indole-deficient” GF and mutant *E. coli* monocolonized mice. (*A*) Representative images of DCX-DAB–stained immature neurons in the DGs of SPF and GF male mice. The black dashed boxes indicate the comparative areas that are magnified to show the notable increase in DCX^+^ neurons. (*B*) DCX^+^ immature neuron populations are significantly reduced in the DGs of GF (*n* = 7) compared with SPF (*n* = 13) male mice. (*C*) Male and female GF mice were inoculated with WT *E. coli* *tnaA* or mutated *E. coli* *ΔtnaA* and their progeny maintained in a controlled environment until experimental testing. (*D*) WT and MT *E. coli* indole production was qualitatively assessed by Kovac’s assay, whereby the presence of indole is indicated by the presence of a pink color change in the alcohol layer of the reaction mixture. (*E*) Liquid chromatography–mass spectrometry analysis showing concentration of indole acetic acid is significantly reduced in serum of MT *E. coli* compared with WT *E. coli* male mice (*n* = 10/group). (*F*) Representative images of DCX-DAB–stained immature neurons in the DGs of WT *E. coli* and MT *E. coli* mice. The black dashed boxes indicate comparative areas that are magnified for clarity. (*G*) DCX^+^ immature neuron populations are significantly reduced in the DGs of MT *E. coli* mice (*n* = 8) compared with WT *E. coli* male mice (*n* = 5). (*H*) Representative images of DCX-DAB–stained immature neurons in the DGs of GF male mice treated with sham or indole-supplemented drinking water (200 μM) for 5 wk. The black dashed boxes indicate comparative areas that are magnified for clarity. (*I*) DCX^+^ immature neuron populations are significantly increased in the DGs of male GF mice supplemented with indole (*n* = 4) compared to vehicle drinking water (*n* = 9). (*J*) Representative images of DCX)-DAB–stained immature neurons in the DGs of MT *E. coli* male mice treated with sham or indole-supplemented drinking water (200 μM) for 5 wk. The black dashed boxes indicate comparative areas that are magnified for clarity. (*K*) DCX^+^ immature neuron populations are significantly increased in the DGs of MT *E. coli* male mice supplemented with indole (*n* = 7) compared with vehicle drinking water (*n* = 8). In all images, nuclei are stained with DAPI (blue). (Scale bars: 100 μm.) All data are presented as mean ± SEM. Statistical differences were determined using Mann–Whitney *U* test (*B*, *G*, *I*, and *K*) and Student’s *t* test (*E*). Asterisks indicate a significant difference between groups (****P < 0.0001, **P < 0.01).

To assess the impact of microbiota on AHN in C57BL/6J mice, we contrasted 3,3-diaminobenzidine (DAB) staining against doublecortin (DCX), which labels proliferating mitotic NPCs committed to a neural lineage (57), between the dentate gyri of GF and specific pathogen–free (SPF) mice. In line with recent findings in younger mice of the same strain (58), we observed diminished neurogenesis in GF mice compared with age-matched SPF controls (1,020 ± 40 versus 700 ± 30 DCX^+^ cells for SPF versus GF mice, respectively; *P* ≤ 0.0001; Fig. 1 *A* and *B*).

The BBB is permeable to indole (59, 60), and GF mice display reduced levels of indole derivatives in serum (38). To test whether microbiota-derived indoles modulate neurogenesis, we monocolonized GF mice either with a wild-type *Escherichia coli*^*tnaA+*^ (WT *E. coli* mice) or with *E. coli*^*tnaA-*^ containing a mutated, nonfunctional tryptophanase (*tnaA*) enzyme (MT *E. coli* mice) (61) (Fig. 1*C*). MT *E. coli* mice had lower serum concentrations of indoles compared with WT *E. coli* mice (Fig. 1 *D* and *E*; *P* ≤ 0.001). MT *E. coli* mice also displayed reduced neurogenesis in the DG compared with WT *E. coli* mice (Fig. 1 *F* and *G*; 690 ± 20 versus 510 ± 30 DCX^+^ cells for WT *E*. *coli* versus MT *E. coli* mice, respectively; *P* ≤ 0.0016). Providing indole in drinking water (200 μM) to either “indole-deficient” GF or MT *E. coli* mice for 5 wk increased neurogenesis relative to that of control mice receiving standard drinking water (Fig. 1 *H* and *I*; 720 ± 30 versus 1,005 ± 30 DCX^+^ cells for control versus indole-treated GF mice, respectively; *P* ≤ 0.0028 and Fig. 1 *J* and *K*; 510 versus 630 ± 12 DCX^+^ cells for control versus indole-treated MT *E. coli* mice, respectively; *P* ≤ 0.0037), underscoring the potential role of indole and its derivatives in the control of neurogenesis.

**Fig. 2.**
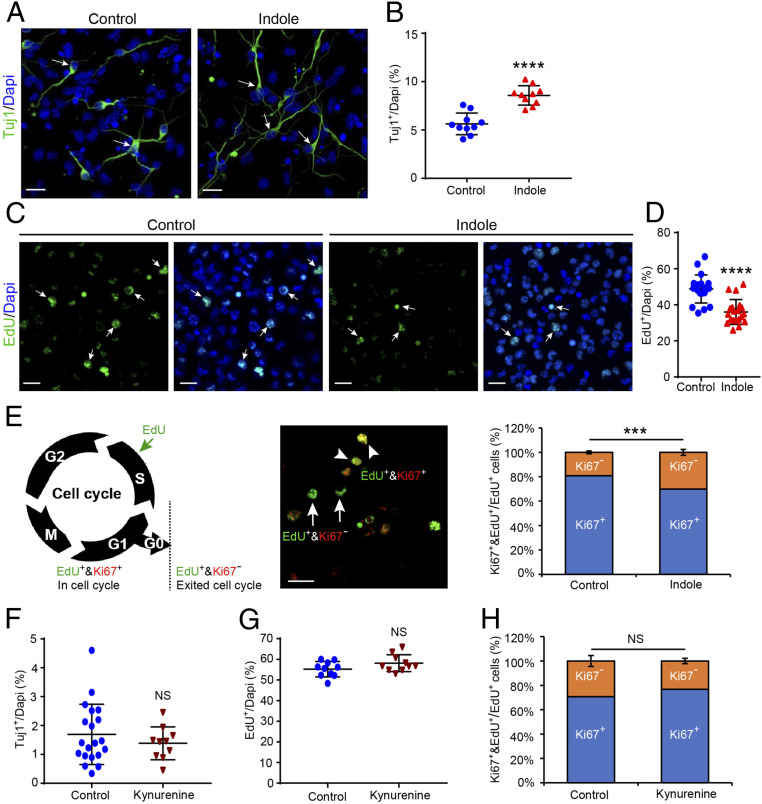
Indole, not kynurenine, exerts neurogenic effects on neuronal progenitor cells ex vivo. (*A*) Representative images of class III Tuj1-immunostained neurons (green) in NPC cultures treated with indole- (100 μM) or vehicle-supplemented media for 4 d. The white arrows indicate Tuj1^+^ neurons. (Scale bar: 20 μm.) (*B*) Quantification of Tuj1^+^ neuron populations reveal that indole treatment enhances neurogenesis (*n* = 10 views/coverslip from *n* = 3 separate NPC cultures). (*C*) Representative images of EdU^+^ cells (green) 24 h after labeling (0.001 µM) and treatment with indole- (100 μM) or vehicle-supplemented media. The white arrows indicate EdU^+^ cells. (Scale bar: 20 μm.) (*D*) Quantification of EdU^+^ cell populations reveal that indole treatment significantly reduces proliferation of NPCs (*n* = 20 views/coverslips from *n* = 3 separate NPC cultures). (*E*, *Left*) Diagram indicating the markers used to assess cell cycle stage. (*Middle*) Representative image of double-labeled EdU^+^&Ki67^+^ cells in cell cycle (arrowhead) and single-labeled EdU^+^&Ki67^−^ cells out of cell cycle (arrow). (Scale bar: 20 μm.) (*Right*) Quantification of EdU^+^&Ki67^+^ versus EdU^+^&Ki67^−^ reveals that indole treatment significantly increases NPC cell cycle exit. (*F–H*) Quantification of (*F*) Tuj1^+^ neuron populations (*G*), EdU^+^ cell populations and (*H*), EdU^+^&Ki67^+^ versus EdU^+^&Ki67^−^ cells in kynurenine- (100 μM) or vehicle-treated NPC cultures shows kynurenine has no effect on NPCs (*n* > 10 views/coverslip from *n* = 3 separate NPC cultures). All data are presented as mean ± SEM. Statistical differences were determined using Mann–Whitney *U* test. Asterisks indicate a significant difference between groups (****P < 0.0001, ***P < 0.001, and NS represents nonsignificant differences).

We next explored whether indole exerted direct neurogenic effects on NPCs using ex vivo neurosphere cultures comprising NPCs and NSCs. NPC proliferation, cell cycle characteristics, and neuronal differentiation were assessed. Indole treatment increased the relative number of progenitors that differentiated into class III beta-Tubulin (Tuj1^+^) neurons compared with vehicle-treated controls (Fig. 2 *A* and *B*; 5.6 ± 0.4% versus 8.6 ± 0.3% for control versus indole-treated NPCs, respectively; *P* ≤ 0.0001). No differences in total cell numbers or programmed cell death reflected by terminal deoxynucleotidyl transferase dUTP nick end labeling (TUNEL) assay were observed (*SI Appendix*, Fig. S1 *A* and *B*). To assess whether changes in NPC proliferation contributed to the observed phenotypes, NPCs were labeled with 5-Ethynyl-2′-deoxyuridine (EdU), a widely used marker of proliferation (62), 1 h before treatment with indole and 24 h prior to staining against EdU/Ki67 (Fig. 2*E*). Neuronal progenitors treated with indole displayed a lower proportion of EdU^+^-labeled cells after 24 h (Fig. 2 *C* and *D*; 49 ± 2% versus 36 ± 2% for control versus indole-treated NPCs, respectively; *P* ≤ 0.0001), but there was an ∼10% increase in EdU^+^/Ki67^−^ cells (Fig. 2 *E*, *Middle* and *Right*; 19 ± 1% versus 30 ± 2% Ki67^−^EdU^+^/EdU^+^ cells for control versus indole-treated NPCs, respectively; *P* ≤ 0.0008) compared with vehicle-treated cells. Thus, indole promotes the exit of NPCs from the cell cycle and commitment to neurogenesis. We then tested whether these effects generalized to all AhR ligands. In contrast to the microbiota-derived indole, treatment with kynurenine—the major tryptophan metabolite of eukaryotic cells—did not induce any changes to the extent of neurogenesis (Fig. 2*F*; 1.7 ± 0.2% versus 1.4 ± 0.2% for vehicle- and kynurenine-treated NPCs, respectively; *P* ≤ 0.64965). Moreover, a slight increase in the proliferation capacity and proportion of NPCs retained in the cell cycle was observed (Fig. 2*G*; 55 ± 1% versus 58 ± 1% for vehicle- versus kynurenine-treated NPCs, respectively; *P* ≤ 0.1655 and Fig. 2*H*; 71 ± 4% versus 77 ± 2% for vehicle- versus kynurenine-treated NPCs, respectively; *P* ≤ 0.4476). No evidence of impaired kynurenine transport into NPCs was found (*SI Appendix*, Fig. S1 *C* and *D*).

**Fig. 3.**
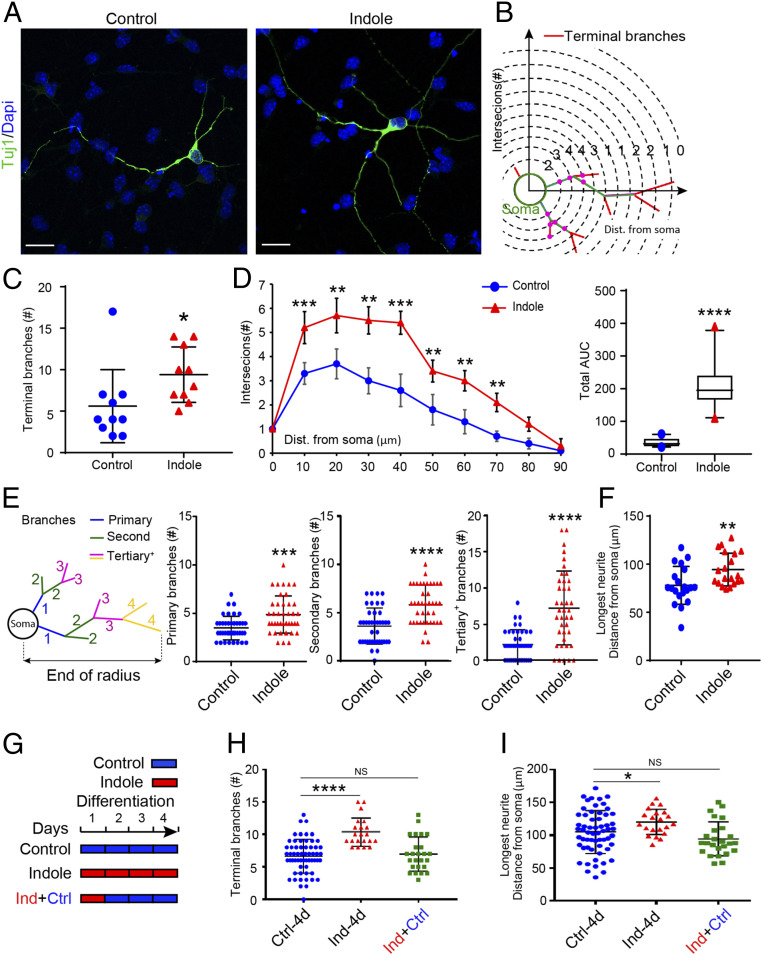
Indole promotes neuronal maturation ex vivo. (*A*) Representative images of Tuj1-immunostained neurons (green) in NPC cultures treated with indole- (100 μM) or vehicle-supplemented media for 4 d. Nuclei (blue) stained with DAPI. (Scale bar: 20 μm.) (*B*) Diagram indicating the quantification of neurite branching by Sholl analysis. (*C* and *D*) Quantification of total number of terminal branches revealed a significant effect of indole to promote neuronal maturation (*D*, *Left*) Sholl analysis quantification of intersections with distance from the cell soma. (*Right*) The corresponding total AUC in the Sholl plot. (*E*) Diagram indicating the quantification of neurite branching by I/O scheme analysis and quantification of neurite branches showing significant increase in primary, secondary, and tertiary in indole-treated NPCs. (*F*) Quantification of longest neurite length revealed a significant increase in neurons differentiated in indole-treated media. (*G*) Timeline for treatment of NPCs with indole (100 μM) at different time points during neuronal differentiation. (*H* and *I*) Quantification of terminal branch number (*H*) and longest neurite length (*I*) revealed that the differentiation of NPCs in indole-supplemented media for 24 h followed by 3 d with vehicle media failed to enhance neuronal maturation (*n* > 10 neurons per coverslip [each circle or triangle for one neuron] from *n* = 3 cultures per treatment condition). Data are presented as mean ± SEM with the exception of *D*, *Right* in which the horizontal line in the box plot represents the mean, and the whiskers show the 10 to 90th percentile. Statistical differences were determined using Mann–Whitney *U* test (*C***–***F*), Kruskal–Wallis test (*H*), and one-way ANOVA (*I*). Asterisks indicate a significant difference between groups (****P < 0.0001, ***P < 0.001, **P < 0.01, *P < 0.05, and NS represents nonsignificant differences).

Further characterization of ex vivo neurospheres revealed that indole promotes neuronal maturation. Neurons differentiated for 4 d either in medium supplemented with indole (100 μM) or vehicle-only medium were identified by Tuj1 immunostaining, and those in fields of comparable cell density (Fig. 3*A*) were analyzed by Sholl (Fig. 3*B*) and “inside out” (I/O)-labeling schemes (as described in ref. 63) (Fig. 3 *E*, *Left*). Semiautomated Sholl analysis showed that indole-supplemented neurons displayed ca. 32% more terminal branches (Fig. 3*C*; 5.6 ± 1 versus 9.4 ± 1 for control versus indole-treated neurons; *P* ≤ 0.0112) and a greater degree of branching along their length (Fig. 3*D*); indole-supplemented neurons had a greater number of primary, secondary, and tertiary branch points (Fig. 3*E*; 3.5 ± 0.2 versus 4.9 ± 0.3 primary branches, *P* ≤ 0.0003; 3.6 ± 0.3 versus 5.9 ± 0.3 secondary branches, *P* ≤ 0.0001; and 2.3 ± 0.3 versus 7.3 ± 0.8 tertiary branches, *P* ≤ 0.0001 for control versus indole-treated neurons, respectively). Moreover, neurons differentiated in indole-supplemented medium had longer neurites (Fig. 3*F*; 78.0 ± 4 μm versus 94.4 ± 4 μm for control versus indole-treated neurons, respectively; *P* ≤ 0.0032). Neurons analyzed after a 24-h exposure to indole followed by differentiation for 3 d in vehicle-only medium (Fig. 3*G*) did not display enhanced maturation (Fig. 3 *H* and *I*).

### Indole Induces Adult Neurogenesis In Vivo.

**Fig. 4.**
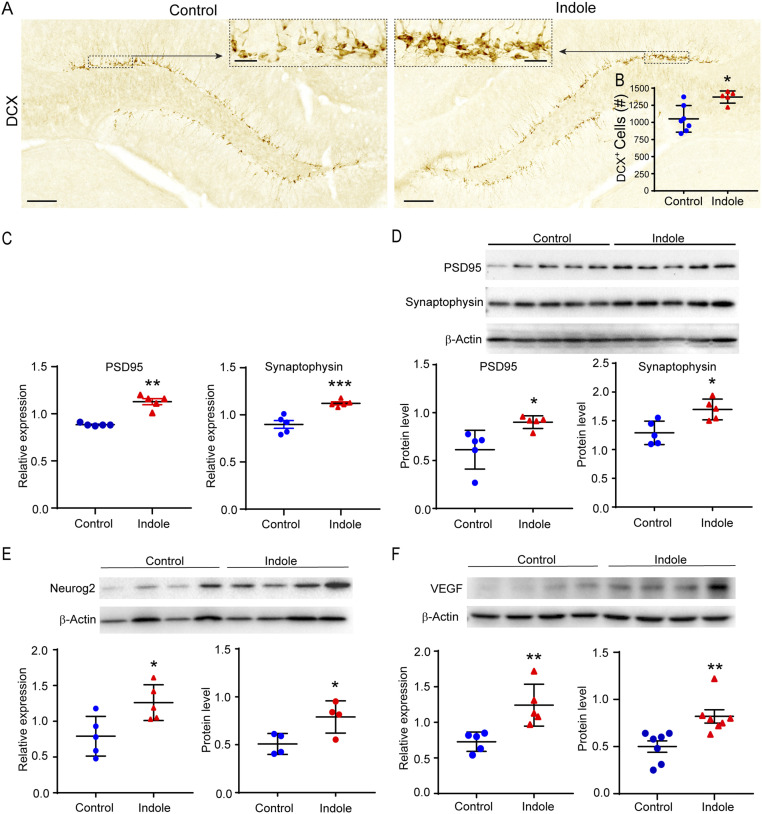
*(A)* Representative images of DCX-DAB stained immature neurons in the DG of SPF mice fed standard or indole-supplemented water (200 μM) for 5 wk. The black dashed boxes indicate comparative areas that are magnified to show the notable increase in DCX+ neurons in indole-supplemented mouse DG. *(B)* DCX+ immature neuron populations are reduced in the DG of mice receiving vehicle- (*n* = 7) compared with indole-supplemented drinking water (*n* = 5). *(C* and *D)* Differential expression of synapse-related genes and proteins in the DG of mice receiving vehicle or indole-supplemented water for 5 weeks. *(C)* PSD-95 and Synaptophysin mRNA (*n* = 5). *(D)* PSD-95 and Synaptophysin proteins (*n* = 5). *(E* and *F)* Differential expression of proteins and genes in the DG of mice receiving vehicle- or indole-supplemented water for 10 days. *(E)* Neurog2 protein (*n* = 7) and mRNA (*n* = 5). *(F)* VEGFa-165 protein (*n* = 7) and mRNA (*n* = 5). The Synaptophysin blot shown in (*D*) was stripped and re-probed with anti-PSD-95. Statistical differences were determined using Mann-Whitney U test (*B* and *F, Right)* or Student’s *t* test (*C–F, Left*). Asterisks indicate a significant difference between groups (***P < 0.001, **P < 0.01, *P < 0.05).

We explored the potential for stimulation of neurogenesis by an oral administration of indole. Supplementing the drinking water of WT C57BL/6J mice with indole (200 μM) for 5 wk increased numbers of DCX^+^ cells in the DG (Fig. 4 *A* and *B*; 1,051 ± 734 versus 1,370 ± 40 for control versus indole-treated mice, respectively; *P* ≤ 0.0177). Indole supplementation did not have any effects on water intake or body weight (*SI Appendix*, Fig. S2 *A* and *B*). Moreover, we found evidence for the functional integration of neurons generated after indole supplementation through an assessment of synapse expression based on the presence of presynaptic synaptophysin (SYP) and postsynaptic density 95 (PSD-95) in the hippocampus (64). Indole-supplemented mice displayed increased *PSD-95* and *SYP* mRNA in the hippocampus compared with controls (Fig. 4*C*; 1.3-fold, *P* ≤ 0.0079 and 1.2-fold, *P* ≤ 0.001, respectively). The expression of these proteins was correlated with similar elevations of PSD-95 and SYP protein levels (Fig. 4*D*; 1.5-fold and 1.3-fold, respectively, *P* ≤ 0.05). The expression of genes associated with synaptic function/plasticity, including AMPAR *GluA1* subunit, vesicular glutamate transporter (*vGlut2*) and calmodulin kinase II (*CaMKII*) were increased in the hippocampus of indole-exposed mice (*SI Appendix*, Fig. S3 *A–D*; 1.7-fold *P* ≤ 0.07, 1.5-fold *P* ≤ 0.05, and 1.5-fold *P* ≤ 0.05 compared with control mice). *VEGF* mRNA and protein were both increased by indole supplementation (Fig. 4*F*; *P* ≤ 0.01). In addition, of the receptor tyrosine kinases through which VEGF signals [VEGFR1, VEGFR2, and neuropilin-1 (NRP1) (21)], *VEGFR2* and *NRP1* mRNA were up-regulated (*SI Appendix*, Fig. S3 *E* and *G*; 1.5-fold, *P* ≤ 0.05, *P* ≤ 0.01, respectively, compared with control mice). We also found increased expression of the proneural basic helix transcription factor Neurogenin-2 (*Neurog2*) at both the mRNA and protein level (Fig. 4*E*; 1.6-fold, *P* ≤ 0.05 and 1.8-fold, *P* ≤ 0.05, respectively). *VEGF* and *Neurog2* are downstream targets of the Wnt/β-catenin pathway, which promotes different stages of adult neurogenesis [reviewed by Varela-Nallar and Inestrosa (65)]. We therefore probed additional targets in this pathway by RT-PCR and discovered elevated Wnt3a ligand, frizzled receptor (*Fzd7*), and β-catenin (*Ctnnb1*) transcripts (*SI Appendix*, Fig. S3 *H–J*; 1.5-fold, *P* ≤ 0.01; 1.4-fold, *P* ≤ 0.05; and 1.3-fold, *P* ≤ 0.01, respectively).

### The AhR Is Pivotal For the Neurogenic Effects of Indole.

**Fig. 5.**
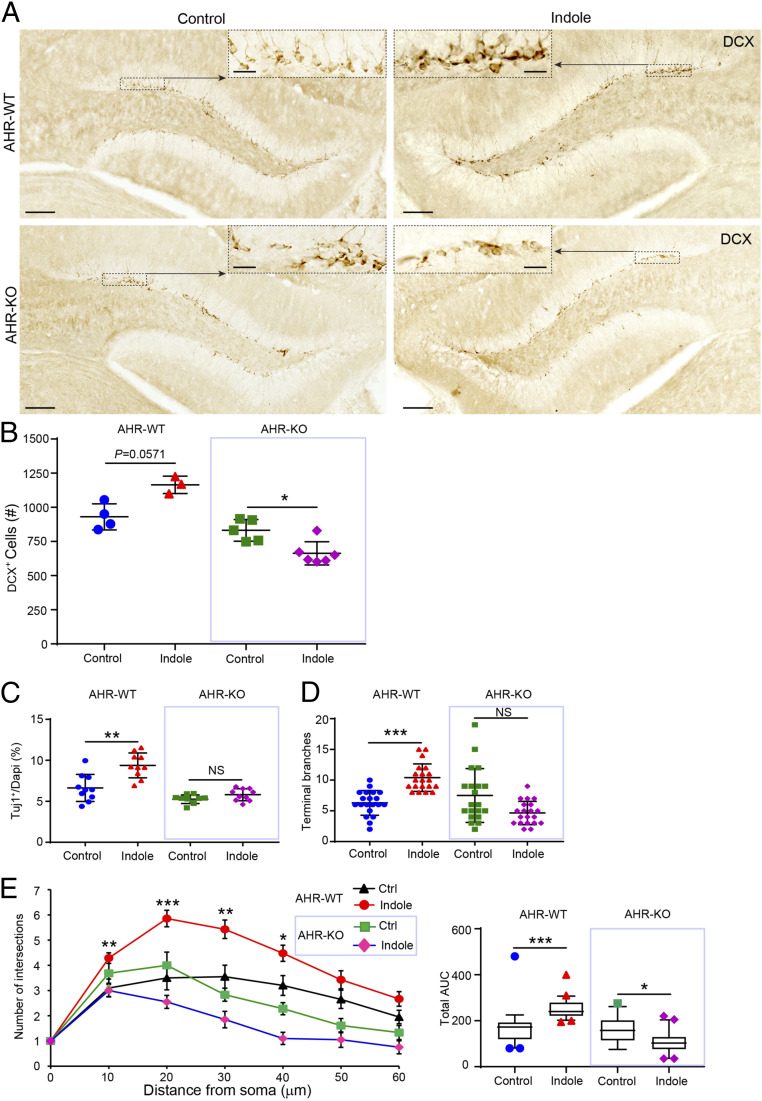
Indole promotes neurogenesis via the AhR ex vivo and in vivo. (*A*) Representative images of DCX-DAB–stained immature neurons in the DGs of SPF mice fed standard or indole-supplemented water (200 μM) for 5 wk. The black dashed boxes indicate comparative areas that are magnified to show the notable increase in DCX^+^ neurons in indole-supplemented AhR-WT but not AhR-KO mouse dentate gyri. (*B*) Quantification of DCX^+^ immature neuron populations in the DGs of vehicle-treated AhR-WT mice (*n* = 4) compared with indole-treated AhR-WT mice (*n* = 3) and vehicle-treated AhR-KO mice (*n* = 5) compared with indole-treated AhR-KO mice (*n* = 6). (*C*) Quantification of Tuj1^+^ neurons revealed indole enhances neurogenesis in AhR-WT NPCs but not AhR-KO NPCs. (*D* and *E*) Quantification of neurite branching revealed a significant effect of indole to promote neuronal maturation in AhR-WT NPCs but not AhR-KO NPCs. (*E*, *Left*) Sholl analysis quantification of intersections with distance from the cell soma (*n* > 10 neurons per coverslip [each circle or triangle for one neuron] from *n* = 3 cultures per treatment condition). (*Right*) The corresponding total AUC in the Sholl plot. Data are presented as mean ± 2 SEM with the exception of *E*, *Right* in which the horizontal line in the box plot represents the mean and the whiskers show the 10 to 90th percentile. Statistical differences were determined using the Kruskal–Wallis test for nonparametric data. Asterisks indicate a significant difference between groups (***P < 0.001, **P < 0.01, *P < 0.05, and NS represents nonsignificant differences).

Indoles activate the AhR signaling pathway. To explore its contribution to indole-dependent neurogenesis, we evaluated the neurogenic potential of indole in AhR-knockout (KO) mice. Indole failed to promote neurogenesis in AhR-KO mice (Fig. 5 *A* and *B*). Moreover, numbers of DCX^+^ cells in the DG of indole-treated AhR-KO mice were lower than in WT controls. Indole also failed to promote neurogenesis in ex vivo neurospheres cultured from AhR-KO mice (Fig. 5 *B–D*) or up-regulate *Neurog2*, *VEGF*, or *β-catenin* transcription in the hippocampus of AhR-KO mice (*SI Appendix*, Fig. S4 *A–C*).

## Discussion

Here, we show that microbiota-derived indole promotes AHN by utilizing a mouse model in which GF mice are monocolonized with *E. coli* capable or incapable of metabolizing tryptophan. Additional data demonstrate that indole supplementation rescues adult neurogenesis in GF mice. This effect appears to be ligand specific for indole, as a different tryptophan metabolite, kynurenine, failed to induce neurogenesis ex vivo. Similar neurogenic effects were also observed in adult C57BL/6J (male) mice treated with indole. Mechanistically, we observed that indole-induced hippocampal neurogenesis is mediated by the AhR signaling pathway since indole treatment failed to increase neurogenesis effects in either AhR-KO mice or AhR-KO ex vivo neurospheres.

Previous studies aimed at assessing the impact of microbes on hippocampal neurogenesis have revealed somewhat differing results, possibly due to different experimental conditions and timelines for monitoring neurogenesis. For instance, in one study, a different mouse line (Swiss Webster) was used (66), whereas in another, hippocampal neurogenesis was assessed earlier in life (58). A third study reported a decrease in adult neurogenesis of adult male C57BL/6J mice after antibiotic treatment (67), which is consistent with our results. Variation in microbiota dynamics and function (35, 68–71) and differences in the energy composition of the chow fed to mice in these studies may account for some of the discrepancies.

We observed increased adult neurogenesis in three rodent models after treatment with indole, demonstrating that mice respond to environmental indoles beyond critical windows of development. A recent report demonstrating increased AHN in 4-wk-old GF mice after either a transplantation of microbes from 24-mo-old mice or a supplementation of diet with the microbiota-derived short chain fatty acid butyrate are consistent with this (72). There now is a need to assess neurogenesis and the effects of indole supplementation in the fetal brain, where indole metabolites are reduced by antibiotic treatment or GF rearing (73).

Our ex vivo studies display direct, ligand-specific neurogenic effects of indole treatment on NPCs. Consistent with previous reports demonstrating anticancer properties of indole derivatives via cell cycle arrest mechanisms (74), we found that the enhancement of neurogenesis ex vivo was not accounted for by elevated proliferation or by a greater survival of neurons but rather by an increased exit of NPC from the cell cycle and differentiation toward a neuronal lineage. Moreover, indole-treated mice display elevated hippocampal neurogenin 2, a bHLH proneural factor that promotes NPC cell cycle withdrawal and neuronal differentiation (75) in addition to neurite outgrowth (76).

Indole-mediated neurogenic effects correlated with increased VEGF-α. Earlier reports have described a promotion of adult neurogenesis (77, 78), greater hippocampal dendritic arborization (79), and neuroprotective and synaptotrophic effects on lesioned neurons with VEGF-α (80). While it remains unclear which receptors transduce VEGF signals in NPCs and neurons, VEGFR2 (also known as FLK1 or KDR)—which was up-regulated at the gene expression level in indole-treated hippocampi—is critical for VEGF-induced enhancements of hippocampal neuron dendritic arborization (79). While our observations and these additional effects of indole may arise directly through interactions with the AhR in NPCs or NSCs, our data do not exclude effects mediated by AhR-expressing bystander cells, for example, astrocytes (40). Further investigations are needed.

To integrate into preexisting circuits and participate in hippocampal functioning, newborn neurons undergo dynamic neurite remodeling resulting in the formation of new synapses (81, 82). Our results demonstrated a profound enhancement of neurite outgrowth upon the treatment of NPCs with indole. We also found increased densities of pre- and postsynaptic markers in vivo, suggesting that indole enhances synaptogenesis.

Kynurenine, the most abundant of all tryptophan metabolites in eukaryotic cells (>90%), also permeates the BBB. In contrast to microbiota-derived indoles, kynurenine exerted no effect on neurogenesis in our study, suggesting ligand specificity in AhR-mediated promotion of adult neurogenesis. Differences in responses to these AhR ligands have been found in other systems. For example, in hyperproliferative cells, kynurenine has been shown to promote tumor growth (83), whereas we have previously reported that indole treatment reduces tumor load via the Wnt/β-catenin pathway in mice (84). Elevated serum kynurenine is often associated with chronic inflammation, accelerated aging, and neurodegenerative disease in humans (49), whereas higher indole concentrations are associated with an extended health span in flies, worms, and rodents (85) and an extended lifespan of bats (86).

In humans, ∼10% of the microbiota population can metabolize tryptophan to generate tryptophan metabolites, suggesting individual variation (34). Therefore, are individuals with a higher content of indole-producing microbes “better off” to respond to environmental stress conditions and thus impose more effective neuroprotective mechanisms? If so, maintaining gut microbiota production of indoles, for example, by dietary intervention regimes and/or exercise-induced microbial stress, may exert “beneficial” effects on the aging host, including promotion of adult neurogenesis in the hippocampus.

The indole-AhR signaling pathway is one mechanism through which gut microbes promote AHN. Given that systemic indole levels decline with age in humans (87) and have been reported to promote greater health and life spans in animal models, our work highlights the potential for translational applications of indole supplementation as part of intervention strategies to slow cognitive decline in neurodegenerative diseases or to promote neural regeneration or repair of the brain or spinal cord after injury.

## Materials and Methods

### Animals.

All experiments were performed in accordance with institutional guidelines and approved by the Regional Animal Research Ethical Board, Institutional Animal Care and Use Committee, Singapore (protocol nos. AUP-E0025 and 2016/SHS/1263). To assess the effects of indole on AHN in vivo, adult (10- to 14-wk-old) male mice on a C57BL/6J genetic background were used. GF, SPF, monoassociated *E. coli*^*tnaA+*^ (WT *E. coli*), and *E. coli*^*tnaA-*^ (MT *E. coli*) mice and AhR^+/+^ and AhR^−/−^ male mice were used as described, and the number of mice used per experiment is indicated in the figure legends. GF and monoassociated mice were bred, experimentally manipulated, and maintained inside contained isolators that were analyzed weekly for contaminants by plating fecal homogenates on agar plates. SPF mice were housed in air-conditioned isolated cages. All mice were provided with an autoclavable rodent diet 5010 (LabDiet) and water ad libitum and maintained with 12-h light/dark cycles. Growth was monitored weekly using a digital scale accurate to two decimal places. Ex vivo studies were performed to evaluate the direct effects of indole on NPCs. For this purpose, adult SPF female mice were timed mated. Briefly, mice were paired overnight, and E0.5 was assigned as the morning a vaginal plug was noted. Pregnant dams were monitored for weight gain daily until embryo harvesting at E14.5.

### GF and SPF Mice.

#### *E. coli* monoassociated mice.

The WT *E. coli* BW25113 strain and the single-gene *tnaA* BW25113δ KO mutant were obtained as a gift from T. K. Wood (Pennsylvania State University, University Park, PA), distributed in sterile vials, and transferred into isolators housing GF mice. C57BL/6J GF mice were colonized with either *E. coli*^*tnaA+*^ (WT *E. coli* mice) or *E. coli*^*tnaA−*^ (MT *E. coli* mice) by oral gavage (450 μL) culture at optical density of one per mouse, pelleted, and resuspended in 100 μL sterile phosphate-buffered saline (PBS) twice in total (7 d apart). The male offspring of gavaged mice were used for experiments. Indole production by WT or MT *E. coli* was verified using Kovacs reagent (Sigma). Mice colonized with different strains were maintained in separate isolators for the duration of the experiment.

#### AhR-KO mice.

C57BL/6J AhR^−/−^ mice were originally obtained from CLEA Japan, Inc. and AhR^+/−^ breeders obtained by genetic modification of blastocysts as previously described (84). AhR^+/−^ mice were crossbred to produce AhR^−/−^ AhR ^+/−^ and AhR^+/+^ mice used for experiments, which were maintained under SPF conditions and genotyped from tail samples on weaning at 3 wk old.

#### Indole administration.

Male C57BL/6J mice (8- to 12-wk-old SPF, GF, and MT-*E. coli*) were randomly assigned regular drinking water or indole-spiked drinking water. Indole (878.6 mg) was dissolved in milliQ water (500 mL, 15 mM) by stirring for 12 h before being filtered twice and diluted in drinking bottles (final concentration of 200 μM). All water was provided ad libitum and changed weekly. No change in water intake or body weight was observed. Mice were euthanized by carbon dioxide inhalation and tissues harvested at 10 d or 5 wk time points for Western blot and qRT-PCR and immunohistochemistry analysis, respectively.

#### Tissue collection.

Briefly, following blood collection from the heart and intracardial perfusion with PBS, brains were rapidly removed and cut down the midline. The left side of each brain was postfixed in paraformaldehyde (PFA, 4%) for 24 h and cryoprotected in sucrose solution (30%) for 24 h before being frozen in optimal cutting temperature solution (Tissue-Tek). Free-floating cryosections (30 μM) were collected using a freezing microtome (Leica #CM3050S Leica) and stored in cryoprotectant at 4 °C until use. Every 12th section was selected for immunohistostaining. The right half of each brain was micro-dissected in ice-cold PBS and whole hippocampi snap frozen in liquid nitrogen and stored at −80 °C. Serum was separated by centrifugation of whole blood and stored at −80 °C.

#### Metabolite analysis.

Kynurenine and indole-3-acetic acid were quantified using protocols based on previously described methods (88) (*SI Appendix*, *Materials and Methods*).

#### DAB staining, imaging, and newborn neuron quantification.

Free-floating brain sections were washed in PBS (0.01 M) and incubated overnight with monoclonal DCX antibody (1:1,000) at room temperature before incubation with biotinylated goat anti-mouse IgG as per the manufacturer’s instructions (1:1,000; Vector Laboratories). Brain sections were mounted on glass slides (Matsunami, MAS-GP, S9901 76 × 26 mm). DCX-positive immunostaining was visualized by the peroxidase method and DAB kit (SK-4100, Vector Laboratories). The quantification of DCX-labeled cells in the DGs was performed as previously described (89) using a Zeiss Axioscan.Z1 slide scanner microscope by a trained researcher blind to the treatment group. Further details are provided in *SI Appendix*, *Materials and Methods*.

#### RNA extraction and quantitative real-time PCR.

Total RNA was isolated from whole hippocampi using RNeasy Mini Kit (Qiagen) as per the manufacturer’s instructions. RNA purity was confirmed using a Nanodrop 2000 (Thermo Fisher Scientific) and quality determined by gel electrophoresis and visualized on a Bioanalyzer (Agilent). RNA (500 ng) was reverse transcribed using iScript II (Bio-Rad) and the resulting complementary DNA (50 ng) used for quantitative real-time PCR on a Quantstudio 6 Flex Real-Time PCR system using fast SYBR Green PCR Master Mix (Applied Biosystems) and 0.45 μg oligonucleotide pairs (*SI Appendix*, Table S1). Samples were run in triplicates, and biological sample sizes are stated in figure legends. Relative gene expressions were normalized to β-actin and computed by the −2ΔCT method.

#### Western blotting.

Whole hippocampi tissue homogenates (10 μg per lane) were analyzed by Western blot using the following antibodies: anti-PSD-95 (Cell Signaling Technology, #3409S), anti-synaptophysin (Cell Signaling Technology, #4329S), anti-Neurog2 (Cell Signaling Technology, #13144), anti-VEGFα (Abcam, ab68334), or anti-β-actin (Santa Cruz). Protein band quantification was performed by densiometry analysis against β-actin using ImageJ software (NIH). Further details are provided in *SI Appendix*, *Materials and Methods*.

### Ex Vivo Studies.

#### Neurosphere culture.

NPCs were isolated from E14.5 mouse forebrains as previously described (90). Briefly, subventricular zones were dissected and the meninges removed before being digested in Accutase cell detachment solution (STEMCELL Technologies; 100 μL, 30 min at 37 °C) followed by mechanical dissociation by trituration with a Pasteur pipette. Single cells were resuspended at 40,000 cells per mL in NPC proliferation medium (EmbryoMax with L-Glutamine, without Hepes Dulbecco’s Modified Eagle Medium/F12 [Sigma-Aldrich, Merck]) supplemented with recombinant fibroblast growth factor-2 (20 ng ⋅ ml^−1^, Invitrogen), human epidermal growth factor (10 ng ⋅ mL^−1^) B-27, and *N*-2 (1%, 100×) (all growth factors are from Gibco, Thermo Fisher Scientific). Neurosphere cultures were passaged upon growth to a diameter of 100 to 200 μm (typically every 3 to 4 d) and single cell NPCs from generations one to three used for experiments at the time of passage.

#### NPC ligand treatment.

To assess the effects of tryptophan ligands on proliferation potential, NPCs (2 × 10^5^ cells) were seeded in wells containing 5-ethynyl-2′-deoxyuridine (EdU)-supplemented NPC proliferation media (Click-iT EdU Cell Proliferation Kit, Alexa Fluor 488 dye, Invitrogen, Thermo Fisher Scientific). Ligand stock solutions (dissolved in minimum volume dimethyl sulfoxide [DMSO] and diluted in NPC proliferation medium) were added to NPCs to varying final concentrations. DMSO vehicle was used as a control. Following incubation (12 h, 37 °C, 5% CO_2_), cells were digested with Accutase (10 min, 37 °C) and single cells (100 μL, ca. cells) seeded onto glass coverslips coated with Poly-L-lysine (0.002%, Sigma-Aldrich, Merck) and laminin (STEMCELL Technologies). Following cell adherence (2 h, 37 °C, 5% CO_2_), coverslips were fixed with PFA (4%, 30 min, room temperature) and stored in PBS at 4 °C until immunostaining.

To assess differentiation potential, NPCs (2 × 10^5^ cells) were plated onto laminin-coated coverslips as previously described and incubated in wells containing varying concentrations of tryptophan ligands or DMSO diluted in NPC medium (500 μL). After 4 d incubation (37 °C, 5% CO_2_) cells were fixed in PFA and stored in PBS as above. The same protocol was used to examine neuronal morphology, except a lower density of cells (1 × 10^5^ cells) were plated on day 0.

#### NPC immunostaining.

Cells were permeabilized and nonspecific binding sites blocked (30 min in 1% bovine serum albumin, 0.1% Triton-X in PBS) and stained by immunofluorescence with anti-Ki67 (1:500, Abcam) or anti-βΙΙΙ tubulin (1:500, Abcam) primary antibodies overnight and visualized with Alexa 488– (green) or Alexa 568– (red) conjugated secondary antibodies (1:400, Invitrogen). Nuclear counterstaining was performed using DAPI (0.25 μg/uL Sigma-Aldrich, Merck). EdU labeling and TUNEL staining was conducted as following the manufacturers protocols (Thermo Fisher Scientific). Coverslips were imaged at 40× magnification using a Zeiss LSM 980 Confocal Laser Scanning microscope. Images (30 per coverslip, three coverslips per experimental group) were acquired of every field of vision from top to bottom along the center line of each coverslip. Images were analyzed using the cell counter and Simple Neurite Tracer functions in ImageJ (Fiji, Image J, NIH). An additional assessment of neurite branching was conducted by manual counting using the I/O-labeling scheme previously described (63). Further details are provided in *SI Appendix*, *Materials and Methods*.

#### Statistics.

All continuous variable data were assessed for normality using the Shapiro–Wilk test using GraphPad Prism version 9.1.0. Parametric data were analyzed by Student’s *t* test for comparisons between two groups or by one-way ANOVA for comparisons between more than two groups. Nonparametric data were analyzed by the Mann–Whitney *U* test for comparisons between two groups or the Kruskal–Wallis test for comparisons between more than two groups. Area under the curve (AUC) for the number of neurite intersections with distance from the soma were calculated for Sholl profiles using built-in AUC analysis in GraphPad Prism software. The details of statistical tests used are provided in the figure legends. *P* values ≤ 0.05 were considered significant.

## Supplementary Material

Supplementary File

## Data Availability

All study data are included in the article and/or *SI Appendix*.
