# Supplementary material for: Tryptophan-metabolizing gut microbes regulate adult neurogenesis via the aryl hydrocarbon receptor
Source: Proc Natl Acad Sci U S A. 2021 Jul 1;118(27):e2021091118. doi: 10.1073/pnas.2021091118 (PMC8271728; doi:10.1073/pnas.2021091118)
Supplement: Supplementary File [file pnas.2021091118.sapp.pdf]

PNAS
www.pnas.org

Supplementary Information for
Tryptophan metabolizing gut microbes regulate adult neurogenesis *via* the aryl hydrocarbon receptor

George Zhang Wei,[a,b,1], Katherine. A. Martin,[a,b,1], Peter Yuli Xing,[c,d], Ruchi Agrawal,[c], Luke Whiley,[e,f], Thomas K. Wood,[g], Sophia Hejndorf,[h], Yong Zhi Ng,[i], Jeremy Zhi Yan Low,[a], Janet Rossant,[j], Robert Nechanitzky,[k], Elaine Holmes,[e,l], Jeremy K. Nicholson,[e,m], Eng-King Tan,[b], Paul M. Matthews,[a,n,o,p,*] and Sven Pettersson,[a,b,h,q,**]

Sven Pettersson
Email: Sven.Pettersson@ki.se

This PDF file includes:

Supplementary text
Figures S1 to S4
Tables S1

SI Materials and Methods

DAB staining, imaging and newborn neuron quantification

Free floating brain sections were washed in PBS (0.01 M) and incubated overnight with monoclonal doublecortin (DCX) antibody (1:1,000) at room temperature before incubation with biotinylated goat anti-mouse IgG as per the manufacturer's instructions (1:1000; Vector Laboratories, Burlingame, CA) in blocking solution at room temperature. Brain sections were mounted on glass slides (Matsunami, MAS-GP, S9901 76 x 26 mm) such that spatially similar sections from all experimental groups were represented on the same slide. Following washing with PBS, slides were incubated with streptavidin-conjugated HRP (Mouse on mouse kit, PK-2200, Vector Laboratories) for 2 hours. DCX-positive immunostaining was visualized by the peroxidase method and diaminobenzidine (DAB) kit (SK-4100, Vector Laboratories) as previously described. Quantification analysis was performed by a trained researcher blind to treatment group using a Zeiss Axioscan.Z1 slide scanner microscope. Total number of DCX labeled cells in the hippocampal dentate gyrus were counted as previously described (1).

Western blotting

Whole hippocampi were lyzed by mechanical homogenization in radioimmunoprecipitation assay (RIPA) buffer containing Complete Protease and Phosphatase inhibitors (Roche, Mannheim, Germany). Protein content of lysates was estimated using Bradford Assay (Biorad) as per the manufacturer's instructions. Protein samples (10 μg per lane) were reduced in Laemmli buffer (Biorad) supplemented with β-mercapthoethanol with heating (98 °C, 5 minutes) prior to separation by SDS-PAGE. Following protein transfer, non-specific binding sites of PDVF membranes (Biorad) were blocked in 5% bovine serum albumin (Sigma) dissolved in Tris.HCl buffer containing 0.1 Tween-20 for 1 hour at room temperature and probed with the following primary antibodies (all 1:1000 in 5% BSA in TBST) overnight, with gentle shaking at 4 °C: anti-PSD-95 (Cell Signaling Technology, #3409S), anti-synaptophysin (Cell Signaling Technology, #4329S), anti-Neurog2 (Cell Signaling Technology, #13144), anti-VEGFα (Abcam, ab68334) or anti-β-actin (Santa Cruz). Immuno-detection with an appropriate horseradish peroxidase (HRP)-conjugated antibody (DAKO) was followed by enhanced chemiluminescence (Clarity ECL, Biorad). Protein band quantification was performed by densiometry analysis against β-actin using ImageJ software (NIH, Bethesda, MD, USA).

In Vivo Metabolite Analysis.

In brief, samples were first spiked with 20 μL of stable isotope labelled internal standards followed by protein precipitation with 250 μL of methanol containing 10 mM ammonium formate. Samples were then vortex mixed and transferred to a solid phase extraction plate (Phenomenex PHREE plate, Phenomenex, Macclesfield UK). PHREE plates were then washed with an additional 250 μL of methanol containing 10 mM ammonium formate. The collection plate was then dried under nitrogen overnight. Dried extracts were re-

suspended in 100 µL containing 10 mM ammonium formate and 0.5% formic acid. LC-MS quantitative analysis was performed using a Waters Acquity UPLC coupled to a Waters Xevo TQ-S MS (Waters Corp., Wilmslow, UK).

<u>LC-MS settings</u>
The LC instrument setup consisted of a Waters Acquity UHPLC LC system and a Waters 2777C external autosampler (Waters, Wilmslow, U.K.). Chromatographic separation was performed using a Waters HSS T3 2.1 × 150 mm, 1.8 µm column (Waters, Wilmslow, U.K.). The mobile phase was composed of 0.1% formic acid in water (v/v) (A) and 0.1% formic acid in acetonitrile (v/v) (B). The column temperature was maintained at 45 °C. The linear gradient was performed as in the table below:

Time	% phase A	% phase B	Flow rate (ml/min)
0	99	1	0.6
3	90	10	
4	10	90	
4.1	99	1	
7	99	1	

MS detection was performed with a Waters Xevo TQ-S tandem quadrupole instrument (Waters, Wilmslow, U.K.) using positive electrospray ionization (ESI). Nitrogen was used as the desolvation gas, and argon was used as the collision gas. The following generic source conditions were used in positive ionization mode: capillary voltage, 2.5 kV; source offset, 30 V; desolvation temperature, 600 °C; source temperature, 150 °C, desolvation gas flow, 1000 L/h; cone gas flow, 150 L/h; nebulizer gas, 7.0 bar; collision gas, 0.15 mL/min.

***Ex Vivo* Metabolite Analysis.**

<u>Metabolite extraction</u>

<u>Cell Pellets</u>
Cell pellets were spiked with 10 µL of water containing Kynurenine-d4 internal standard. Cells were then lysed using 50 µL of methanol, followed by 5 minutes of sonication and 10 minutes of vortex mixing. The extracts were then centrifuged. 60 µL of supernatant was removed and transferred to a 96 well plate. Finally 190 µL of water was added to each well to dilute the final sample. Plates were sealed, vortex mixed prior to injection onto the LC-MS system.

<u>LC-MS method</u>
Samples were analysed using a Waters Acquity LC system coupled to a Waters Xevo TQ-S mass spectrometer. Separation was completed using a Waters BEH C18 column (50mm x 2.1mm x 3µm). Mobile phase A consisted of water (0.1% formic acid) and acetonitrile (0.1% formic acid). The gradient started at 25% B an remained constant for 0.1 minutes, increasing to 95% B at 0.5 minutes, returning immediately to 25% B and holding until 1 minute.

Two mass spectrometry MRM transitions were monitored for each metabolite to facilitate accurate identification, except for the internal standard that utilised a single MRM transition. Kynurenine (positive ionisation mode, 209.1 > 94.1, 209.1 >146.1); indole (positive ionisation mode, 118.1 > 65.1, 118.1 > 91.1); indoxyl sulfate (negative ionisation mode, 212.1 > 80.12, 212.1 >132.4); kynurenine-d4 (positive ionisation mode, 213.1 >150.1).

Data Processing

Data was processed in SkylineMS v20.2. Peak areas for each target were generated in SkylineMS, prior to further processing in R using in house scripts.

NPC immunostaining

Cells were permeabilized and non-specific binding sites blocked (30 minutes in 1% BSA, 0.1% Triton-X in PBS) and stained by immunofluorescence with anti-Ki67 (1:1000, Abcam) or anti-βIII tubulin (1:1000, Abcam, Cambridge, UK) primary antibodies overnight and visualized with Alexa 488 (green) or Alexa 568 (red)-conjugated secondary antibodies (1:400, Invitrogen). Nuclear counterstaining was performed using 4', 6-diamidino-2-phenylindole (DAPI) (0.25 ug/uL Sigma-Aldrich, Merck). EdU labeling and TUNEL staining was conducted as following the manufacturers protocols (ThermoFisher Scientific, MA) followed by incubation with nuclear Hoechst stain. Coverslips were mounted on glass slides with Prolong Gold Antifade Mountant (5 uL, Invitrogen, ThermoFisher Scientific, MA). Coverslips were imaged at 40X magnification using a Zeiss LSM 980 Confocal Laser Scanning microscope. Images (30 per coverslip, 3 coverslips per experimental group) were acquired of every field of vision from top to bottom along the centre-line of each coverslip. The laser strength of each channel (DAPI, 488 nm, and 594 nm) was adjusted to optimize the visualization of stains between experimental runs. The images were analyzed using the Fiji distribution of ImageJ. The number of proliferating (EdU^+), apoptotic ($TUNEL^+$) and total ($DAPI^+$) cells was counted automatically using an adapted ImageJ batch processing macro. For quantitative analysis, single or double-labeled cells were counted using the cell counter function in Fiji, ImageJ. To assess neuronal morphology, images (20 representative mature neurons per coverslip) were traced and Sholl analysis conducted (inter-ring distance set to 10 μm) using the semi-automated Simple Neurite Tracer tool (Fiji, Image J, NIH, Bethesda, MD, USA). Additional assessment of neurite branching was conducted by manual counting, using the "inside-out" labeling scheme previously described (2).

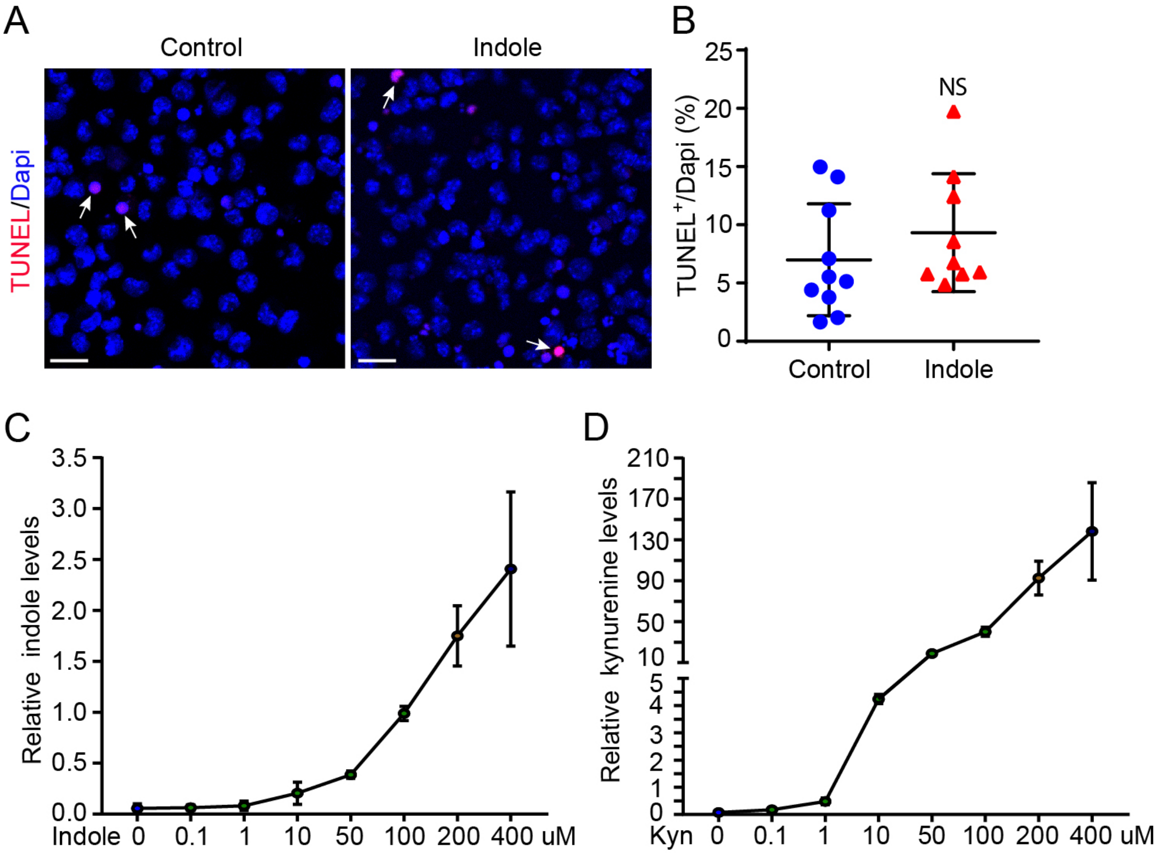

Fig. S1. Indole has no effect on cell death *ex vivo* (*A*) Representative images of TUNEL (red) and Dapi (blue) stained cells in NPC cultures treated with vehicle or indole (100 μM) for 24 hours. Scale bar: 20 μm. **(*B*)** Quantification of $TUNEL^{+}$/Dapi cells reveals no difference between vehicle and indole-treated NPCs (n = 10 views/coverslip from n = 3 separate NPC cultures). **(*C—D*)** Semi-quantitative mass spectrometry analysis of NPCs treated with increasing concentrations of (***C***): indole and (***D***): kynurenine, reveal dose-dependent accumulation in the cell fractions. Data are presented as Mean ± SEM. Statistical differences were determined using Mann-Whitney U test. *NS* represents no statistical significance.

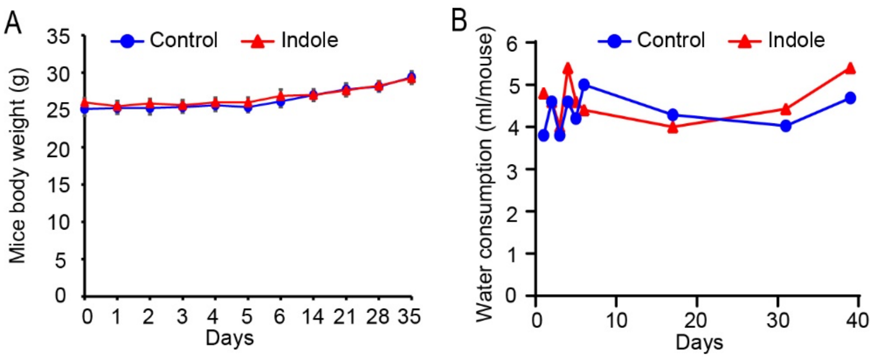

Fig. S2. Exogenous indole has no effect on mouse "general health" parameters (*A* – *B*) Quantification of **(*A*)** mouse body weight and **(*B*)** water intake revealed no changes over the 5 week treatment period (Repeated measures ANOVA with Tukey's test, n = 5). Data are presented as mean ± SEM.

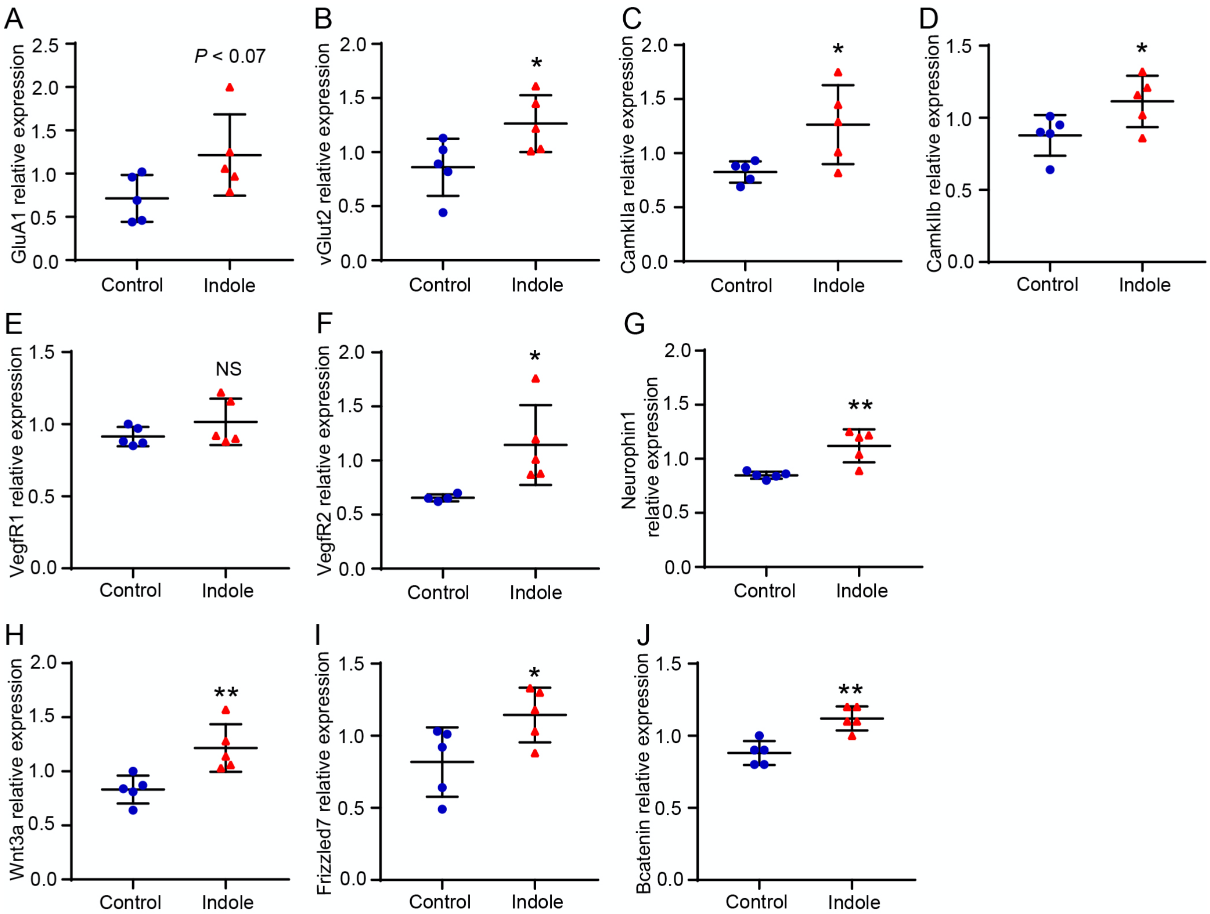

Fig. S3. Differential gene/protein expression in the hippocampus of SPF mice treated with exogenous indole. (A — D) Elevated gene expression of (***A***) *GLUA1* (***B***) *vGLUT2* (***C***) *CamkIIa* (***D***) *CamkIIb* in the hippocampi of mice receiving indole-supplemented drinking water for 10 days, compared to vehicle-control (*$P < 0.05$, Student's *t* test, n = 5). **(E — G)** Differential gene expression of (***E***) *VegfR1* (***F***) *VegfR2* (***G***) *NPLN1* in the hippocampi of mice receiving indole-supplemented drinking water for 10 days, compared to vehicle-control (*$P < 0.05$, **$P < 0.01$, Student's *t* test, n = 5). **(H— J)** Differential gene expression of (***H***) *Wnt3a* (***I***) *Fzd7* (***J***) *β-Catenin* in the hippocampi of mice receiving indole-supplemented drinking water for 10 days, compared to vehicle-control (*$P < 0.05$, **$P < 0.01$, Student's *t* test, n = 5).

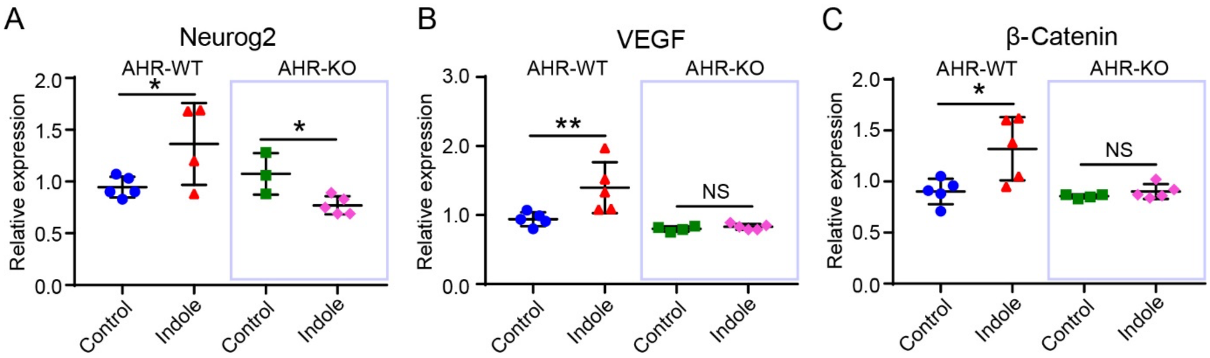

Fig. S4. Indole promotes *Neurog2*, *VEGF* and *β-Catenin* gene expression *via* AhR *in vivo*.

(***A—C***) Differential expression of genes in the hippocampus of AhR-WT or AhR-KO mice receiving vehicle or indole-supplemented water for 10 days. (***A***) *Neurog2* mRNA (***B***) *VEGF* mRNA (***C***) *β-Catenin* mRNA (* $P < 0.05$, ** $P < 0.01$, Student's t test, n = 3-5).

Supplementary Table S1 List of primers used in this paper

Gene	Primer	Sequence (5'-3')
β-Actin	forward	CTGTATTCCCCTCCATCGTG
	reverse	CCTCGTCACCCACATAGGAG
PSD-95	forward	TGAGATCATAGCAGCTACT
	reverse	CTTCCTCCCCTAGCAGGTCC
SYP	forward	TGGTGTTCGGCTTCCTGAA
	reverse	GCGGCCCAGCCTGTCT
Neurog2	forward	AACTGGAGTGCCTTGGAGTC
	reverse	CGAGTCTCGTGTGTTGTCGT
Vegfa	forward	AGGAGGAGGGCAGAATCATCA
	reverse	CTCGATTGGATGGCAGTAGCT
GluA1	forward	ACCCTCCATGTGATCGAAATG
	reverse	GGTTCTATTCTGGACGCTTGAG
vGlut2	forward	TGGTGCAGTACACTGGATGG
	reverse	CGTCTGTTATGGTTGGATGC
Camk2a	forward	CCTGTATATCTTGCTGGTTGGG
	reverse	TTGATCAGATCCTTGGCTTCC
Camk2b	forward	TCAAGCCCCAGACAAACAG
	reverse	TTCCTTAATGCCGTCCACTG
VegfR1	forward	TGGACCCAGATGAAGTTCCC
	reverse	GCGATTTGCCTAGTTTCAGTCT
VegfR2	forward	AGCACTGGTCCTATGGGTTG
	reverse	GGTTCTGCCATTTGATCCA
NRP1	forward	CGTGGAAGTAATTGATGGGGAG
	reverse	CATAGCGGATGGAAAACCCTG
Wnt3a	forward	TCGGAGATGGTGGTAGAGAAA
	reverse	CGCAGAAGTTGGGTGAGG
Fzd7	forward	GCCTCTGTTCGTCTACCTC
	reverse	GTCGTGTTTCATGATGGTGC
Ctnnb1	forward	ATGGAGCCGGACAGAAAAGC
	reverse	CTTGCCACTCAGGGAAGGA

1. C. Y. Tsai, C. Y. Tsai, S. J. Arnold, G. J. Huang, Ablation of hippocampal neurogenesis in mice impairs the response to stress during the dark cycle. *Nat. Commun.* **6**, 1–7 (2015).
2. C. G. Langhammer, *et al.*, Automated Sholl analysis of digitized neuronal morphology at multiple scales: Whole cell Sholl analysis versus Sholl analysis of arbor subregions. *Cytom. Part A* **77 A**, 1160–1168 (2010).
